# Staged hybrid procedure versus radiofrequency catheter ablation in the treatment of atrial fibrillation

**DOI:** 10.1371/journal.pone.0205431

**Published:** 2018-10-09

**Authors:** Jin Kyung Hwang, Dong Seop Jeong, Hye Bin Gwag, Kyoung-min Park, Joonghyun Ahn, Keumhee Carriere, Seung-Jung Park, June Soo Kim, Young Keun On

**Affiliations:** 1 Division of Cardiology, Department of Medicine, Veterans Health Service Medical Center, Seoul, Korea; 2 Division of Thoracic Surgery, Samsung Medical Center, Sunkyunkwan University School of Medicine, Seoul, Korea; 3 Division of Cardiology, Department of Medicine, Samsung Medical Center, Sunkyunkwan University School of Medicine, Seoul, Korea; 4 Biostatistics and Clinical Epidemiology Center, Research Institute for Future Medicine, Samsung Medical Center, Seoul, Korea; 5 Department of Mathematical and Statistical Sciences, University of Alberta, Edmonton, Alberta, Canada; University of Minnesota, UNITED STATES

## Abstract

The treatment effect of the hybrid procedure, consisting of a thoracoscopic ablation followed by an endocardial radiofrequency catheter ablation (RFCA), is unclear. A total of 117 ablation-naïve patients who underwent either the staged hybrid procedure (n = 72) or RFCA alone (n = 105) for drug-refractory, non-valvular persistent or long-standing persistent atrial fibrillation (AF) were enrolled. The primary outcome is occurrence of total atrial arrhythmia, defined as a composite of AF, sustained atrial tachycardia (AT), and atypical atrial flutter (AFL) after index procedure. The mean age was 52.7 years. Eighty-four percentage of the patients were male. Patients with prior history of stroke and long-standing persistent AF were more prevalent in the hybrid group than RFCA group. The left atrial volume index was larger in the hybrid group (P<0.001). During 2.1 years of median follow-up, the incidence of total atrial arrhythmia was not different between the two groups (32.5% vs. 35.7%; adjusted hazard ratio: 0.64; 95% confidence interval: 0.36–1.14; P = 0.13). The AF recurrence was significantly lower in the hybrid group than in the RFCA group (29.6% vs. 34.9%; adjusted HR: 0.53; 95% CI: 0.29–0.99; P = 0.046). The hospital stay was longer in the hybrid group than in the RFCA group (11 days vs. 4 days; P<0.001). A staged hybrid procedure may be an alternative choice for drug-refractory persistent AF, but it is no more effective than RFCA alone to eliminate atrial arrhythmias. Considering the long-length of stay and the morbidity, careful consideration should be given in selection of treatment strategy.

## Introduction

Atrial fibrillation (AF) is the most common cardiac arrhythmia, and is associated with increased mortality and morbidity due to outcomes such as stroke or exacerbation of heart failure.[1] A number of non-pharmacological treatment modalities, such as radiofrequency catheter ablation (RFCA) and Cox-maze surgical ablation, have been introduced over the past 20 years to overcome the unsatisfactory success rates of pharmacological treatment.[2] The efficacy of RFCA in treating AF depends on the disease stage, and additional linear ablation or ablation of complex fractionated electrograms could not improve the success rate in persistent AF.[3–5] The original cut-and sew Cox-maze III procedure resulted in long-term freedom from symptomatic AF, but concerns over adverse procedural events remained.[6] Recently, surgical ablation for AF has shifted toward minimally invasive thoracoscopic ablation. Although thoracoscopic ablation achieves higher success rates in the initial study,[7] a recent larger and long-term follow-up study showed conflicting results.[8] Moreover, the thoracoscopic ablation cannot always guarantee contiguous and transmural lesion, and the increase in complications has been still considered as a limitation.

A staged hybrid procedure consisting of the sequential combination of thoracoscopic ablation and RFCA is an attractive alternative that complements the respective limitations of epi- and endocardial approaches. Recent European guidelines have suggested that combination or ‘hybrid’ rhythm control therapy seems to be a reasonable option for treating AF.[9] However, there are limited data regarding the treatment efficacy of the hybrid procedure. Moreover, previous studies have used small patient populations with mixed subtypes of AF and included only short-term clinical outcomes, the results of which have been conflicting. Therefore, we conducted this study to evaluate the long-term treatment effects of a staged hybrid procedure compared with RFCA alone in patients with each AF subtype (persistent and long-standing persistent).

## Materials and methods

### Study population

**Fig 1:**
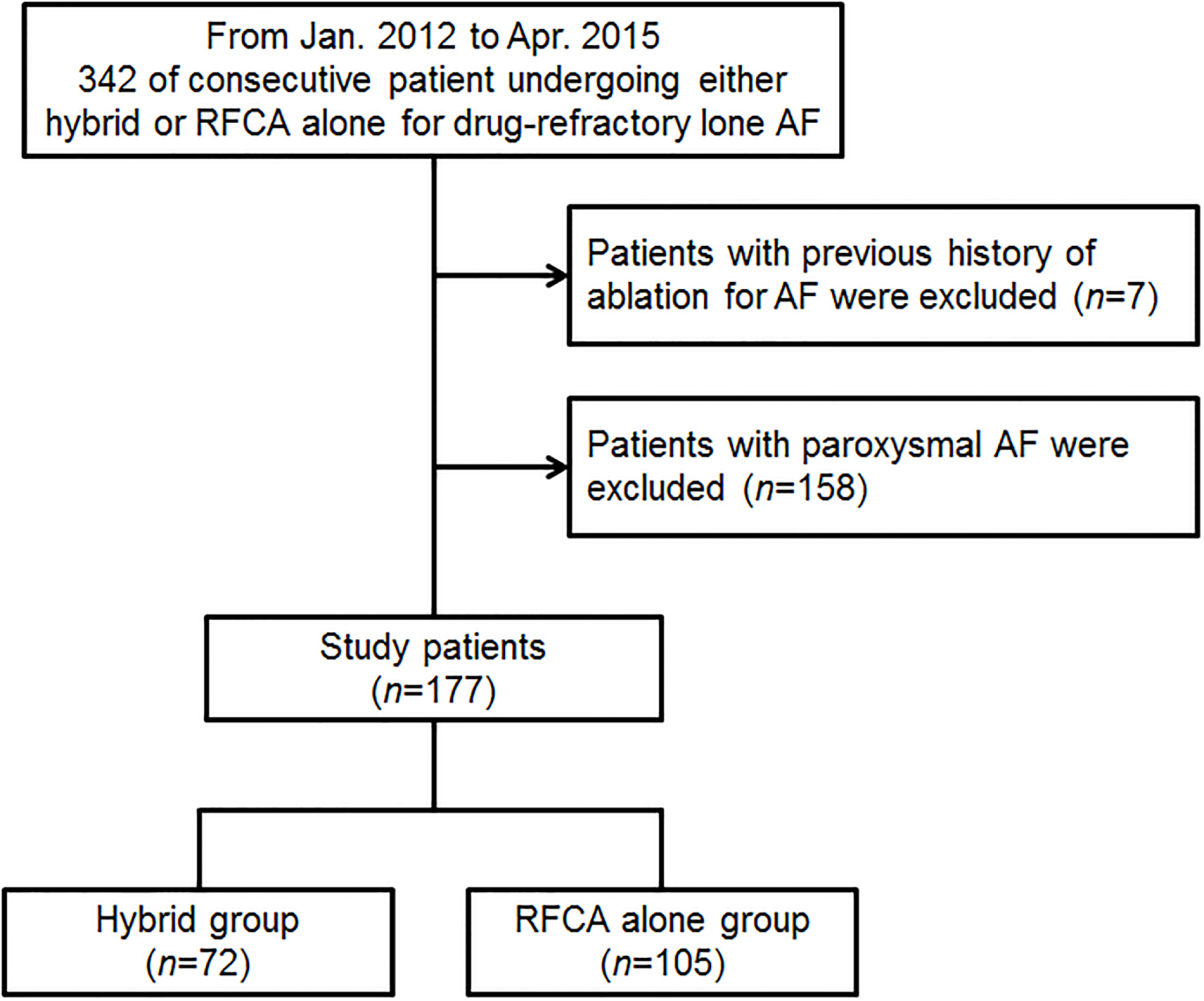
Study scheme. AF, atrial fibrillation; RFCA, radiofrequency catheter ablation.

We reviewed the records of all patients who underwent either a staged hybrid procedure or RFCA alone for symptomatic drug-refractory non-valvular AF at the Samsung Medical Center between January 2012 and April 2015. All patients had been prescribed at least one anti-arrhythmia drug for more than six weeks and had experienced failed medical treatment. At our center, patients who had a history of stroke, preference for a surgical approach, a left atrial (LA) diameter >50 mm, a previous failed catheter ablation, or a relative contraindication to warfarin therapy are preferentially selected for the staged hybrid procedure with concurrent LA appendage resection. After exclusion of patients who had prior history of ablation for AF, a total of 177 ablation-naïve patients were analyzed in this study (Fig 1). All patients underwent transthoracic and transesophageal echocardiography, as well as cardiac computed tomography before the index procedure. Baseline characteristics, and angiographic and procedural findings were collected retrospectively, and data on clinical outcomes were collected prospectively from our AF registry by research coordinators. Further information was collected from medical records or via telephone contact, as necessary. The Institutional Review Board of the Samsung Medical Centre approved this study and waived the requirement for written informed consent for access to an institutional registry (IRB 2012-10-090).

### Surgical techniques

**Fig 2:**
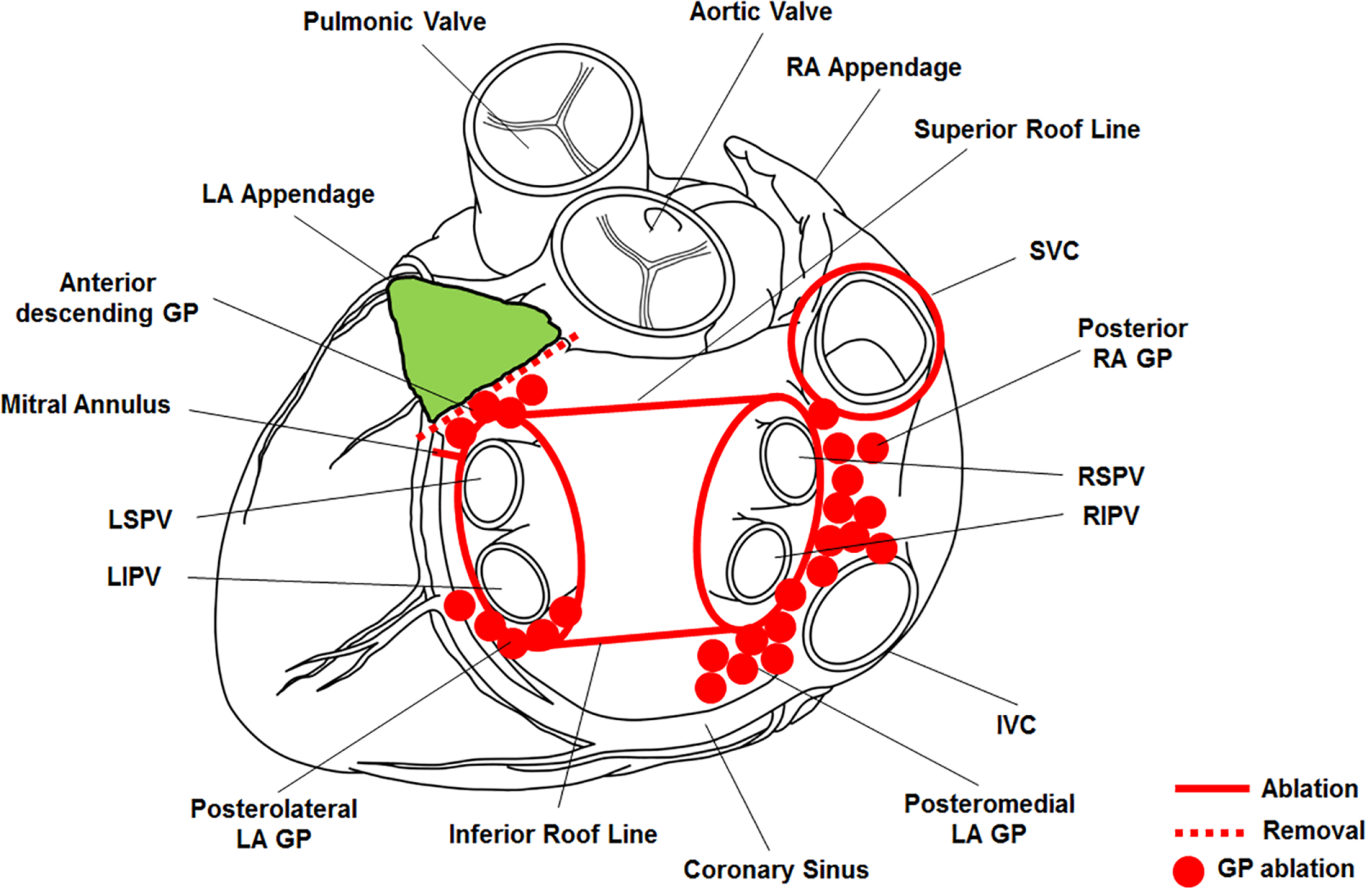
Hybrid procedure with ablation lesions. GP, ganglionated plexus; IVC, inferior vena cava; LA, left atrium; LIPV, left inferior pulmonary vein; LSPV, left superior pulmonary vein; RA, right atrium; RIPV, right inferior pulmonary vein; RSPV, right superior pulmonary vein; SVC, superior vena cava.

The hybrid procedure was performed sequentially, starting with a totally thoracoscopic ablation followed by an electrophysiology study four or five days later to stabilize the high bleeding tendency that can occur immediately after surgery.[10] All procedures were performed using standard techniques as described previously.[2,10] The bilateral thoracoscopic approach was used to make a box lesion and for the LA appendage resection. Each side required only three incisions for one 10-mm port and two 5-mm ports. Right-side procedure was performed first. One 5-mm port was introduced into the fourth intercostal space at the mid-axillary line. The remaining 5-mm port was placed at the third intercostal space of the anterior axillary line, and the 10-mm port was placed at the sixth intercostal space of the mid-axillary line. Carbon dioxide insufflation was used to depress the diaphragm and obtain the intrapericardial operative space. Through the oblique sinus, we used a lighted dissector (AtriCure Lumitip Dissector, AtriCure, Inc., Cincinnati, OH, USA) to pass a rubber band under the pulmonary vein (PV) antrum after pericardial tenting. Then, an AtriCure Isolator Transpolar Clamp (AtriCure, Inc.) was connected to the rubber band and located antrum of PV. We applied six to eight rounds times of bipolar radiofrequency energy to the clamps around the PV antrum according to signals in the control box. Superior and inferior ablation lines forming box line were made by connecting bilateral PV isolation lines using a linear pen device (AtriCure, Inc.) epicardially. Confirmation of the ablation lines via an exit block test was performed by pacing using a flexible AtriCure Cooltip pen (AtriCure, Inc.) at all PVs (superior → anterior → inferior → posterior, respectively). A superior vena cava circular lesion was made using a bipolar clamp in the enlarged right atrium on intraoperative transoesophageal echocardiogram under anesthesia (S1 Fig). The ganglionated plexi (GPs) were ablated according to high-frequency stimulation in the epicardial fat pad anterior side of the right superior and inferior PVs, inferior side of the right inferior PV and the LA posterior wall. A positive high-frequency stimulation response was defined as ≥50% increase in the R-R interval. Using a bipolar ablation pen (Isolator Transpolar pen), the high-frequency stimulation was delivered (cycle length 60 ms, 16 Hz, pulse width 1.0 ms) and output incrementing from 1 to 25 mA. When the high-frequency stimulation did not evoke a vagal response, ablation was performed on the basis of anatomic landmarks. On the left side, GPs in the fat pads on the LA roof, medial to the left superior PV, and inferior side of the left inferior PV were also identified and ablated. Additional GP ablation was applied when necessary. After the PV and GPs ablations, dissection and ablation of the ligament of Marshall was done. Once all of the ablations were complete and conduction block was confirmed, the LA appendage was removed using an Echelon Flex 60 articulating endoscopic linear stapler (Ethicon Endo-Surgery Inc., Cincinnati, OH, USA) (S2 Fig). The procedure sites of the surgical techniques are shown in Fig 2.

### Catheter-based technique

The catheter-based technique in the hybrid group and the RFCA group was identical. Under general anesthesia, we inserted one 6-F quadripolar electrode catheter percutaneously into the left femoral vein, advanced it using fluoroscopic guidance, and placed it in the right ventricular apex. Using a similar technique, one 7-F duodecapolar catheter was placed at the crista terminalis of the right atrium and coronary sinus via the left femoral vein. After positioning the catheters, we introduced two SL1 sheaths (St. Jude Medical, St. Paul, MN, USA) into the LA through a transseptal puncture using fluoroscopy and blood pressure monitoring. Systemic anticoagulation with intravenous heparin was initiated just after transseptal insertion of the sheaths, and activated clotting time was checked every 30 minutes with a target level of 300~350 sec. We checked a multi-view pulmonary venogram using a standard transseptal sheath. Using the Carto 3 system (Biosense Webster, Diamond Bar, CA, USA), we mapped the LA geometry, confirmed the location of the catheters, and integrated the created image with CT images. One Lasso NAV catheter (Biosense Webster) 15~25 mm in diameter was inserted into the PV, after which we checked the PV potential. In patients who required three-dimensional mapping of the LA, a Thermocool ablation catheter (Biosense Webster) and a Lasso catheter were placed in the LA using single or double transseptal access points. We applied radiofrequency energy to remove residual potentials in the four PV antra. Cavotricuspid-isthmus ablation line was made in patients with long-standing persistent AF or in patients with episodes of atrial flutter before or after surgical ablation. When sinus rhythm was not restored despite ablating residual potentials around the PV antra, we ablated the mitral isthmus, mitral annulus, roof line, septal line, and the antrum of the superior vena cava. After each procedure, all catheters were removed and complete hemostasis was ensured.

### Post-procedural care and follow-up

Patients were monitored in the cardiac intensive care unit for the first 24 hours after the procedure. All patients were followed-up at two weeks, three months, six months and every six months thereafter. At three, six, and 12 months after the index procedure, the 24-hour Holter monitoring and echocardiography were performed to evaluate the heart rhythm and atrial activity. If necessary, electric or chemical cardioversion was done within three months of the blanking period to maintain sinus rhythm. Anticoagulants had prescribed during the first three months in all patients, and attempted to stop thereafter if there was no evidence of recurrence after the procedure. Further administration was at the discretion of the individual cardiologist based on the presence or absence of AF, prior stroke history, and the risk of thromboembolic event based on the CHA_2_DS_2_-VASc score.[11] The prescription of antiarrhythmic drugs was made at the discretion of the physician.

### Outcomes and definitions

The primary arrhythmic outcome was occurrence of total atrial arrhythmia, defined as a composite of AF recurrence, sustained AT, and atypical AFL, after the three-month blanking period. Secondary arrhythmic outcomes were recurrence of AF, sustained episodes of atrial tachycardia (AT) or atypical atrial flutter (AFL). All significant atrial arrhythmia were defined as running more than 30 seconds documented by either electrocardiography or 24-hour Holter monitoring after the blanking period. Procedure-related outcomes consisted of the length of hospital stay, stroke, atrioesophageal fistula, pneumonia, pericardial effusion, procedure-related bleeding, sinoatrial node dysfunction, pacemaker implantation, and additional procedure for the recurrent arrhythmia. Definitions of the AF subtypes (persistent and long-standing persistent AF), success and failure of procedure, and the follow-up methods were based on the Heart Rhythm Society, European Heart Rhythm Association and the European Cardia Arrhythmia Society consensus statement.[12]

### Statistical analysis

Continuous variables are presented as the mean ± standard deviation and compared using independent *t*-tests or Wilcoxon rank sum tests. Categorical variables are described as the number (*n*) and percentage (%) and compared using Pearson χ^2^ or Fisher’s exact tests. The Cox proportional hazard analysis was used for comparing the risks of clinical outcomes between the two groups. As covariates, statistically significant variables on univariate analysis and/or clinically relevant variables were included: age, body mass index, gender, subtypes of AF, and LA volume index (LAVI). Antiarrhythmic drugs prescribed after the procedure were included as time-dependent covariates in a Cox survival analysis. Survival curves were made using Kaplan-Meier estimates of the time to the first event and compared using a log-rank test. In subgroup analysis, we converted the continuous variables into binary variables based on the best cutoff value as calculated by ROC curve analysis. All tests were 2-tailed and a *P* value of <0.05 was considered statistically significant. All analyses were performed with the Statistical Analysis Software package (SAS version 9.2, SAS Institute, Cary, NC, USA).

## Results

### Baseline characteristics

The baseline patient characteristics are described in Table 1. Median interval between surgical epicardial ablation and catheter-based endocardial ablation was 6 (interquartile range [IQR]: 5–8) days. The mean age of all patients was 52.70 ± 9.80 years. The percentage of males was higher in the hybrid group than in the RFCA group (*P*<0.001). Long-standing persistent AF was the dominant AF subtype (66.7%) in the hybrid group. The hybrid group also had a higher prevalence of prior stroke and larger average LAVIs than the RCFA group (*P* = 0.03 and *P* = 0.002, respectively). The Procedure-related baseline characteristics are described in Table 2. PV isolation and PV carina ablation were performed in all patients. The number of roof line, circular ablation of superior vena cava, cavo-tricuspid isthmus ablation were not significantly different in both groups. Division of ligaments of Marshall, GPs ablation, LA appendage removal and line between superior and inferior vena cava were formed only in the hybrid group. See the footnote in Table 2 for a more details of the lines.

**Table 1:** Patient-related baseline characteristics.

Variables	Hybrid (*n* = 72)	RFCA alone (*n* = 105)	*P* value
Age (years)	53.57 ± 8.46	52.02 ± 8.63	0.38
Body mass index (kg/m^2^)	25.29 ± 2.57	25.51 ± 3.09	0.72
Male	71 (98.6)	85 (81.0)	<0.001
Types of AF			<0.001
Persistent AF	24 (33.3)	73 (69.5)	
Long-standing persistent AF	48 (66.7)	32 (30.5)	
Coronary artery disease	0	2 (1.9)	0.52
Congestive heart failure	4 (5.6)	1 (1.0)	0.16
Diabetes mellitus	9 (12.5)	19 (18.1)	0.40
Hypertension	31 (43.1)	32 (30.5)	0.11
Hyperthyroidism	1 (1.4)	2 (1.9)	1.00
Prior stroke	11 (15.3)	5 (4.8)	0.03
Chronic kidney disease	0	2 (1.9)	0.52
LV ejection fraction (%)	59.07 ± 6.48	60.19 ± 6.24	0.14
LA volume index (mL/m^2^)	47.93 ± 14.34	41.60 ± 11.90	0.002
CHA_2_DS_2_-VASc*	1.39 ± 1.00	1.18 ± 0.99	0.14

Values are mean ± standard deviation or *n* (%).

*CHA_2_DS_2_-VASc score was calculated by congestive heart failure, hypertension, age ≥65 years, diabetes, prior vascular disease (e.g. peripheral artery disease, myocardial infarction, aortic plaque), female gets one point, and age ≥75 years, prior stroke or transient ischemic attack or thromboembolism gets two points.

AF means atrial fibrillation; LA, left atrium; LV, left ventricle; RFCA, radiofrequency catheter ablation

**Table 2:** Procedure-related baseline characteristics.

Variables	Hybrid (*n* = 72)	RFCA alone (*n* = 105)	*P* value
PVs isolation	72 (100)	105 (100)	-
PV carina ablation	72 (100)	105 (100)	-
Roof line ablation*	62 (86.1)	94 (89.5)	0.49
Inferior line ablation^†^	72 (100)	-	<0.001
Division of ligament of Marshall	67 (93.1)	-	<0.001
Ganglionated plexus ablation	72 (100)	-	<0.001
LA appendage removal	69 (95.8)	-	<0.001
SVC circular ablation	14 (19.4)	24 (22.8)	0.71
SVC-IVC linear ablation	5 (6.9)	-	0.01
CTI ablation	61 (84.7)	95 (90.4)	0.34
Mitral isthmus line ablation^‡^	63 (87.5)	86 (81.9)	0.40
Other linear ablation^§^	-	16 (15.2)	<0.001

Values are *n* (%).

*Roof line means the line between superior ridge of right and left superior pulmonary vein.

^†^Inferior line means the line between inferior ridge of right and left inferior pulmonary vein.

^‡^Mitral isthmus line means the shortest line from the inferior ridge of left inferior pulmonary vein to the mitral annulus.

^§^Other linear ablation is including anterior line (the shortest line from anterior ridge of left inferior pulmonary vein to mitral annulus) or septal line (from anterior ridge of right inferior pulmonary vein to roof of coronary sinus orifice).

CTI means cavo-tricuspid isthmus; LA, left atrium; PVs, pulmonary veins; SVC, superior vena cava; SVC-IVC, superior vena cava and inferior vena cava.

### Arrhythmic outcomes

The median follow-up duration was 2.1 years (IQR: 1.2–3.2 years). During follow-up period, 81.9% of hybrid group (59 patients) and 85.7% of RFCA alone group (90 patients) were performed 24-hour Holter monitoring (*P* = 0.53). There was no significant difference between the hybrid and RFCA alone groups in terms of occurrence of total atrial arrhythmia (32.5% vs. 35.7%, respectively; adjusted hazard ratio [HR]: 0.64; 95% confidence interval [CI]: 0.36–1.14; *P* = 0.13) (Table 3). The two-year AF recurrence rate was 29.6% (18 patients) in the hybrid group and 34.9% (33 patients) in the RFCA alone group. The AF recurrence was significantly lower in the hybrid group than in the RFCA group (HR: 0.53; 95% CI: 0.29–0.99; *P* = 0.046). The incidences of sustained AT and atypical AFL were not significantly different between the two groups (*P* = 0.72 and *P* = 0.60, respectively).

**Table 3:** Arrhythmia outcomes over 2 years.

	Total population					
	Hybrid (n = 72)		RFCA alone (n = 105)		Adjusted HR†	P value
	1-year	2-year	1-year	2-year	(95% CI)	
Total atrial arrhythmia*	14 (19.9)	20 (32.5)	26 (25.5)	34 (35.7)	0.64 (0.36–1.14)	0.13
AF recurrence	12 (17.1)	18 (29.6)	25 (24.5)	33 (34.9)	0.53 (0.29–0.99)	0.046
Sustained AT	1 (0.01)	2 (0.04)	2 (0.02)	3 (0.03)	1.36 (0.26–7.02)	0.72
Atypical AFL	5 (0.1)	8 (0.1)	8 (0.1)	8 (0.1)	1.31 (0.47–3.65)	0.60

Values are n (%) or hazard ratio (95% confidence interval). The percentages shown are Kaplan-Meier estimates from the intention to treat analysis. Significant *P* value is <0.05. The hazard ratio is for the Hybrid group as compared with the RFCA alone group.

*Total atrial arrhythmia, composite of atrial fibrillation recurrence, sustained atrial tachycardia, or atypical atrial flutter

^†^Relevant covariates considered for analysis were age, BMI, male, types of AF, LA volume index, and antiarrhythmic drugs prescribed after index procedure.

AF means atrial fibrillation; AFL, atrial flutter; AT, atrial tachycardia; CI, confidence interval; HR, hazard ratio; RFCA, radiofrequency catheter ablation.

Among patients who had no arrhythmic event and maintained follow-up, anti-arrhythmia drug was prescribed in 40.0% of Hybrid group and 54.7% of RFCA group at 12-month follow-up (*P* = 0.07) (S1 Table). At this time, anticoagulants were used in 24.6% of Hybrid group and 26.3% of RFCA group (*P* = 0.81). At 24-month follow-up, 24.4% of Hybrid group and 38.7% of RFCA group used anti-arrhythmia drug (*P* = 0.04). Anticoagulants were prescribed in 20.0% of Hybrid group and 24.1% of RFCA group (*P* = 0.67).

### Subgroup analysis

**Fig 3:**
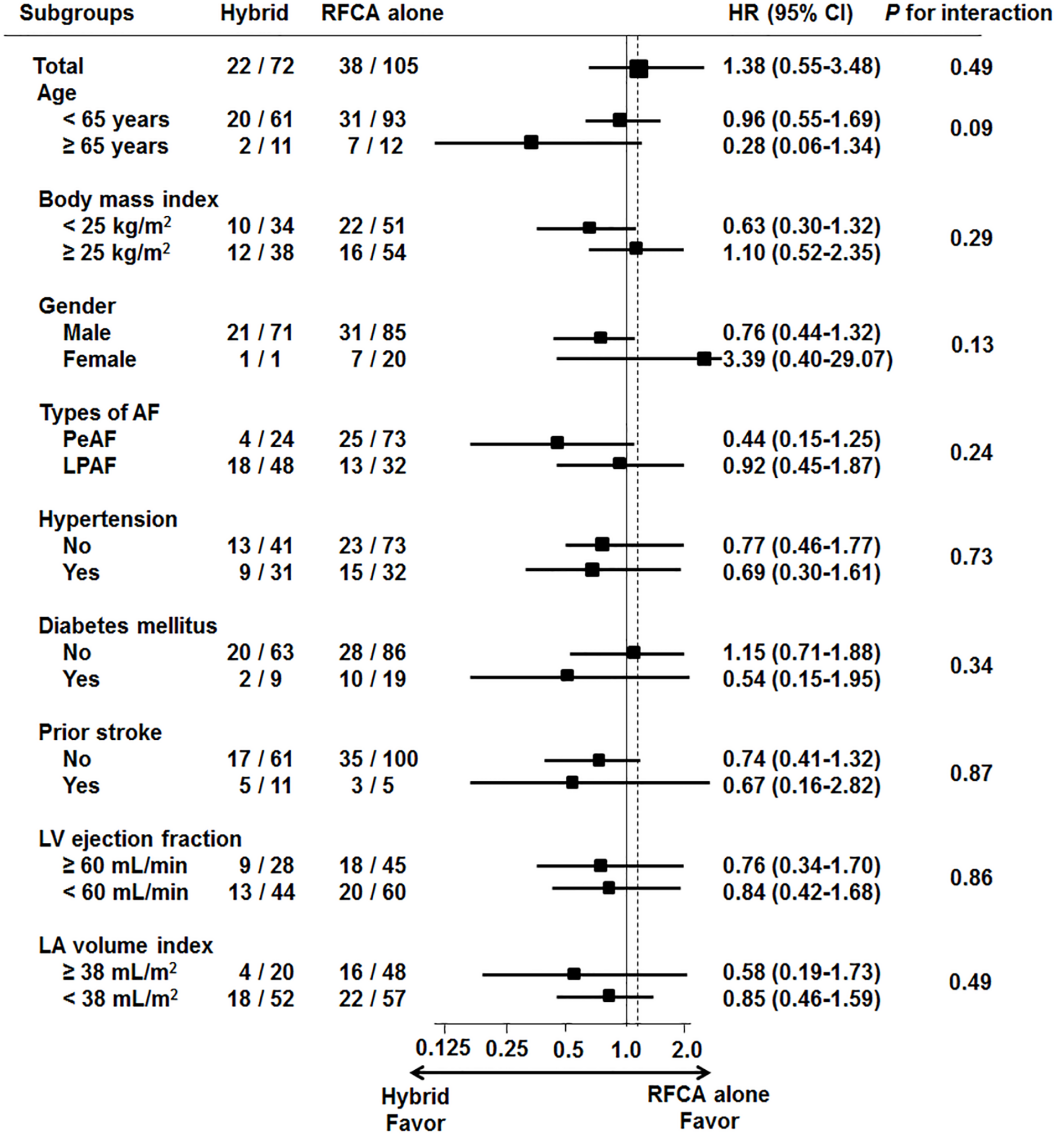
Subgroup analysis for total atrial arrhythmia. AF, atrial fibrillation; HR, hazard ratio; LA, left atrium; LV, left ventricle; PeAF, persistent atrial fibrillation; LSPF, longstanding-persistent atrial fibrillation; RFCA, radiofrequency catheter ablation.

To determine whether the primary outcomes observed in the overall patient population were consistent, we performed post-hoc analyses in various subgroups (Fig 3). The reduction of total atrial arrhythmic burden in the hybrid group was not better than RFCA alone across the overall subgroups.

**Fig 4:**
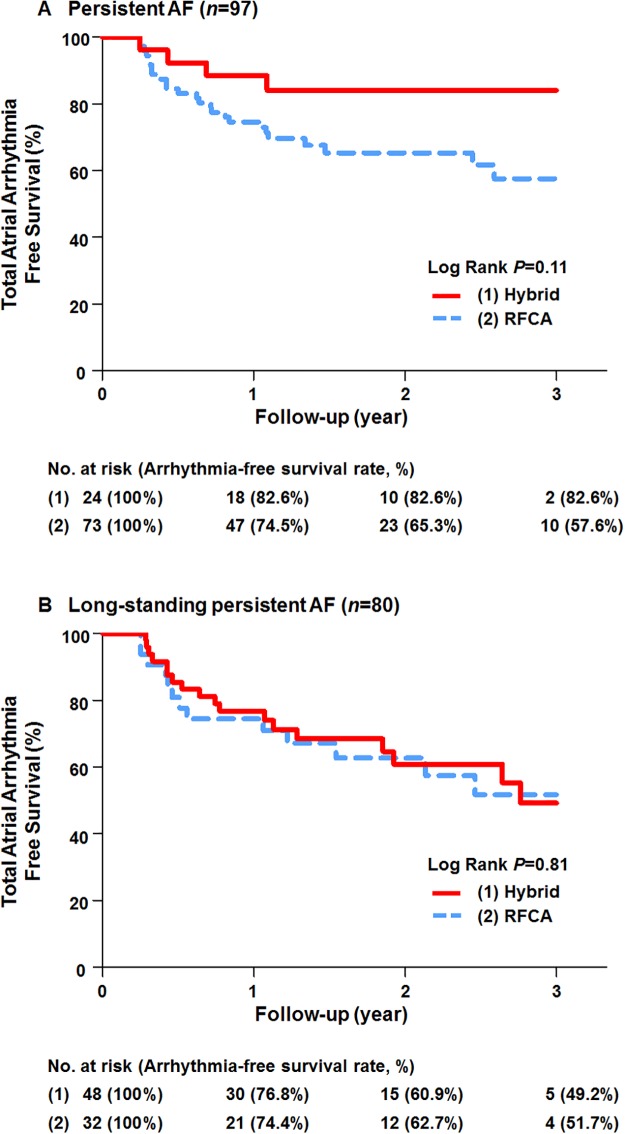
Total atrial arrhythmia-free survival curve by subtype of AF. Comparison of total atrial arrhythmia-free survival rates in patients with (A) persistent AF and (B) long-standing persistent AF. AF, atrial fibrillation; RFCA, radiofrequency catheter ablation.

We compared the total atrial arrhythmia-free survival rate between the hybrid group and the RFCA alone group considering subtype of AF (Fig 4). On the survival curve, the hybrid procedure seems to improve total atrial arrhythmia-free survival rather than RFCA alone in persistent AF, but it was not statistically significant (log-rank, *P* = 0.11). In long-standing persistent AF, there was no significant difference between the two groups for improving total atrial arrhythmia-free survival (log rank, *P* = 0.81).

### Procedure-related adverse outcomes

Procedure-related adverse outcomes are listed in Table 4. No patients died during either procedure or during the follow-up period. Severe potential intraoperative complications, such as conversion to cardiopulmonary bypass, did not occur during the study period. The median hospital stay was significantly longer in the hybrid group than in the RFCA alone group (11 days vs. 4 days, respectively; *P*<0.001). Postoperative stroke was noted in two patients in the RFCA group, but all were remote events that occurred 2~3 years after the procedure. Mild pneumonia occurred in four patients in the hybrid procedure group compared with none in the RFCA only group (*P* = 0.03), but invasive treatments such as ventilator care were not needed. Pericardial effusion was detected in five patients in the hybrid group and two patients in the RFCA group (*P* = 0.12); none of them showed evidence of cardiac tamponade.

**Table 4:** Procedure-related adverse outcomes.

	Hybrid (*n* = 72)	RFCA alone (*n* = 105)	*P* value
Hospital days	11.11 ± 3.28	4.70 ± 1.33	<0.001
Stroke	0 (0.0)	2 (0.8)	1.00
Atrioesophageal fistula	0	0	-
Pneumonia	4 (5.6)	0	0.03
Pericardial effusion	5 (6.9)	2 (1.9)	0.12
Procedure related bleeding	5 (6.9)	2 (1.9)	0.12
Sinoatrial node dysfunction	5 (6.9)	2 (1.9)	0.12
Pacemaker implantation	1 (1.4)	2 (1.9)	1.00
Secondary procedures for recurrence			
Endocardial ablation alone	10 (13.9)	8 (7.6)	0.14
Epicardial ablation alone	0	7 (6.7)	0.01
Redo of hybrid procedure	10 (13.9)	15 (14.3)	1.00

Values are mean ± standard deviation or *n* (%)

### Discussion

In this study, we compared arrhythmic outcomes after a staged hybrid procedure or RFCA alone in patients with drug-refractory AF. The hybrid procedure was associated with better outcomes compared with RFCA alone in terms of AF recurrence after a three-month blanking period over a median follow-up of 2.1 years. However, in the aspect of reduction of total atrial arrhythmia, hybrid procedure had no advantage compared to RFCA alone. Although there was a reduction in AF recurrences with the hybrid procedure, the advantage was offset by an increase in the occurrences of AT and atypical AFL. There were several adverse outcomes occurred after the hybrid procedure, including extension of hospital day and increase in pneumonia or pericardial effusion, but most of the complications were minor.

According to a recent study, the success rate of RFCA is ~80% in paroxysmal AF and ~70% in persistent AF.[13] Recurrence is mainly associated with PV reconnection. However, in some cases multiple procedures, including complex and fractionated atrial electrographic ablation or linear lesions combined with PV isolation, are necessary.[14] Minimally invasive surgical ablation using thoracoscopy is an effective approach for the isolation of the PV antrum and linear ablation of the LA without the risk of thromboembolism. Recently, thoracoscopic ablation was found to be superior to catheter ablation in AF patients with a dilated LA or who had a failed prior catheter ablation, with success rates reported up to 92%.[15–18] Although this approach achieves higher arrhythmia-free success rates, major limitations still remain; successful treatment of transmural lesions cannot be guaranteed and endocardial lesions, such as those made in the mitral isthmus, cannot be created.[19,20] Therefore, the hybrid procedure, a combination of epi- and endocardial ablation, has been expected to improve treatment success rates for AF, especially in patients with persistent- or long-standing persistent AF with a dilated LA.

To date, very few studies have investigated the treatment effects of hybrid procedures, and reported results have been conflicting. Mahapatra *et al*. studied the treatment effects of a sequential hybrid procedure versus repeat RFCA in 45 patients with recurrent long-standing persistent AF.[21] The reported atrial arrhythmia-free survival rates were 86.7% for the hybrid group and 53.3% for the repeat RFCA group. Distinct from our study, all participants of that study had previously undergone RFCA and, therefore, represented a challenging patient population. Edgerton *et al*. compared the success and complication rates of the hybrid and RFCA procedures in 59 patients with long-standing persistent AF and enlarged LAs (>45 mm).[8] In their study, the hybrid procedure showed only a 19% arrhythmia-free survival rate after 24 months of follow-up, significantly inferior to RFCA alone, which had a 54.3% arrhythmia-free survival rate. The hybrid procedure also resulted in a higher rate of complications. In contrast to our study, they used a unipolar RF ablator and simultaneous endocardial ablation strategy, along with an epicardial approach. According to an *in vivo* experimental model, a bipolar clamping device was found to be more helpful than a unipolar device in generating transmural lesions and lead to electrical isolation of the ablated tissue.[19]

In patients with persistent AF, PV isolation could prevent recurrent AF in some, but not all patients. Recently, in the Substrate and Trigger Ablation for Reduction of Atrial Fibrillation Trial Part II (STAR AF II) trial, RFCA AF-free survival rates were at most 59% in patients with persistent AF.[22] Additional ablation strategies should be considered, but currently there is no consensus on the optimal treatment modality for these patients. Our study showed that the hybrid procedure had a better AF-free survival rate compared with that of RFCA alone in patients with persistent AF and long standing-persistent AF. However, considering the occurrence of AT and AFL, the total atrial arrhythmic burden after the procedure was not significantly improved in the hybrid group compared with the RFCA alone group. A few articles have reported incidences of AT or AFL after a hybrid procedure of up to 11%.[23,24] These tachyarrhythmias after ablation are usually caused by macroreentry rotating anatomic barriers such as mitral annulus or prior ablated ostium of PVs. Reconnected PV ostia could induce focal microreentry, it is also cause of tachyarrhythmias after ablation.[25] One of the main causes of our study results in terms of total atrial arrhythmias might be an insufficient procedural interval between epi- and endocardial ablation. Tissue stunning and edema induced by multiple burns during surgery may be associated with an increased rate of false-negative or -positive results during endocardial procedures.[23] Therefore, a sufficient interval for the maturation of ablation lesions makes it easier to detect the boundaries of non-isolated substrates and gap points, and may help to reduce the total atrial arrhythmic burden.

### Limitations

As a non-randomized observational study, there are several limitations in this study. Although we performed statistical methods to adjust potential confounding factors, we could not correct all possible and unmeasured variables. Second, the AF recurrence rates may have been underestimated because we did not include longer heart rhythm monitoring techniques, such as an implantable loop recorder; we only performed 24-hour Holter monitoring due to cost constraints under the current Korean insurance settings. However, we applied the same follow-up protocol in both treatment groups; therefore, this limitation should be equally relevant to both groups. Third, a standardized protocol for the use of medications, including anti-arrhythmia drugs and anticoagulation agents, was absent. Medications were prescribed at the discretion of the physician, which is one of the limitations of a non-randomized study. In addition, very high number of patients receiving anti-arrhythmia drugs in this study. Therefore, there is a limitation to evaluate the procedural success rate in patients without anti-arrhythmia drugs after procedure. However, we tried to overcome these limitations using a time-dependent Cox survival analysis considering prescription of anti-arrhythmia drugs.

### Conclusions

Compared RFCA alone, the staged hybrid procedure could not improve total atrial arrhythmia-free survival during 2-year follow-up period. Although, the hybrid procedure reduced the recurrence rate of AF compared with RFCA alone, the advantage could be offset by an increase in occurrences of AT and atypical AFL after procedure and significantly longer hospital stay, careful consideration should be given in selection of treatment strategy. Further randomized large-scale trials are needed.

## Supporting information

S1 Fig Superior vena cava circular lesion using bipolar clamp. (TIF)

S2 Fig Left atrial appendage removal using an Echelon Flex 60 articulating endoscopic linear stapler. (TIF)

S1 File Prescription of antiarrhythmics and anticoagulants during follow-up period. (DOCX)
