# Supplementary figures and images for: Staged hybrid procedure versus radiofrequency catheter ablation in the treatment of atrial fibrillation
Source: PLoS One. 2018 Oct 9;13(10):e0205431. doi: 10.1371/journal.pone.0205431 (PMC6177159; doi:10.1371/journal.pone.0205431)

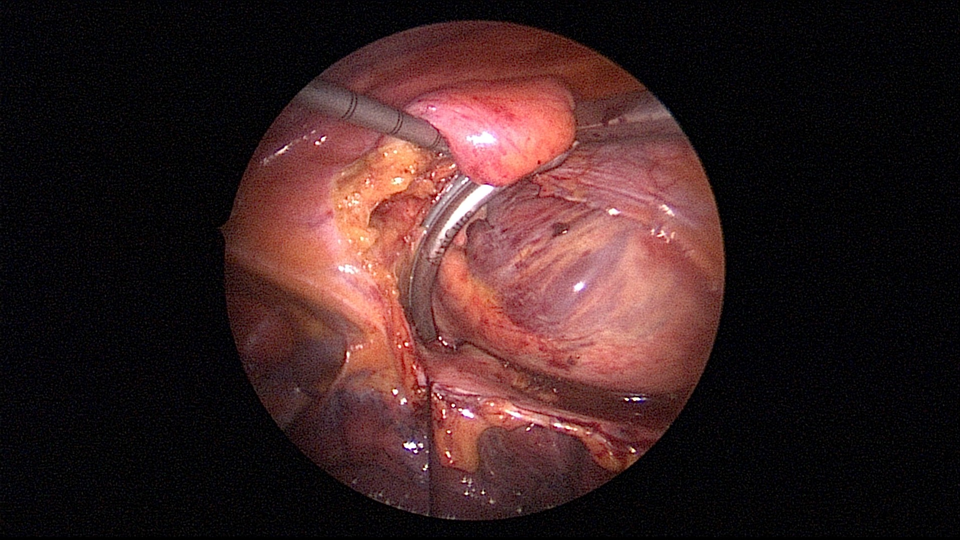

Supplement: S1 Fig — (TIF) [file pone.0205431.s001.tif]

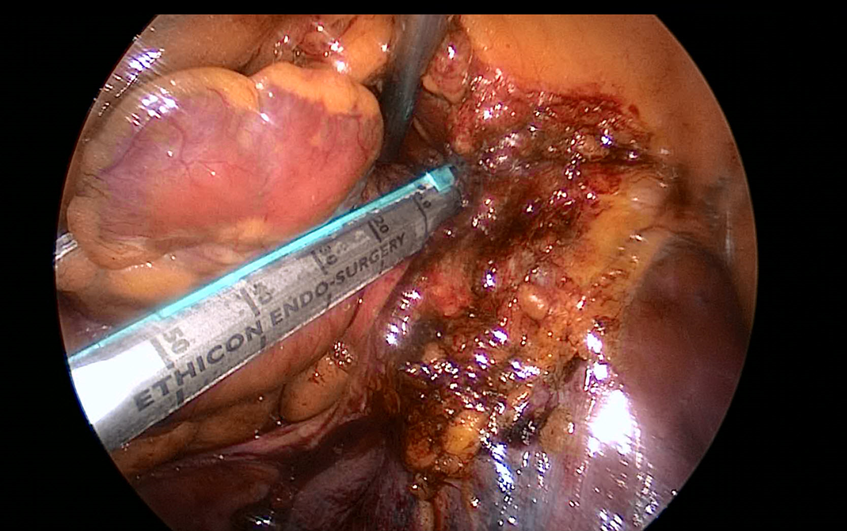

Supplement: S2 Fig — (TIF) [file pone.0205431.s002.tif]
