# Supplementary material for: Staged hybrid procedure versus radiofrequency catheter ablation in the treatment of atrial fibrillation
Source: PLoS One. 2018 Oct 9;13(10):e0205431. doi: 10.1371/journal.pone.0205431 (PMC6177159; doi:10.1371/journal.pone.0205431)
Supplement: S1 File — (DOCX) [file pone.0205431.s003.docx]

**S1 Table.** Prescription of antiarrhythmics and anticoagulants during follow-up period

|  | Hybrid | RFCA alone | *P* value |
| --- | --- | --- | --- |
| 3-month follow-up | (*n* = 72) | (*n* = 105) |  |
| Antiarrhythmics | 56 (77.8) | 82 (78.1) | 1.00 |
| Class Ic | 7 (9.7) | 33 (31.4) | 0.001 |
| Class III | 50 (69.4) | 52 (49.5) | 0.01 |
| Anticoagulants | 38 (52.8) | 39 (37.1) | 0.046 |
| 6-month follow-up | (*n* = 72) | (*n* = 105) |  |
| Antiarrhythmics | 45 (62.5) | 70 (66.7) | 0.63 |
| Class Ic | 5 (6.9) | 26 (24.8) | 0.002 |
| Class III | 40 (55.6) | 45 (42.9) | 0.126 |
| Anticoagulants | 25 (34.7) | 26 (34.7) | 0.18 |
| 12-month follow-up | (*n* = 65) | (*n* = 95) |  |
| Antiarrhythmics | 26 (40.0) | 52 (54.7) | 0.07 |
| Class Ic | 3 (4.2) | 18 (18.9) | 0.001 |
| Class III | 23 (35.3) | 34 (35.7) | 1.00 |
| Anticoagulants | 16 (24.6) | 25 (26.3) | 0.81 |
| 24-month follow-up | (*n* = 45) | (*n* = 62) |  |
| Antiarrhythmics | 11 (24.4) | 24 (38.7) | 0.04 |
| Class Ic | 2 (4.4) | 9 (14.5) | 0.01 |
| Class III | 9 (20.0) | 15 (24.2) | 0.72 |
| Anticoagulants | 9 (20.0) | 15 (24.1) | 0.67 |

Values are *n* (%). NOAC means non-vitamin K antagonist oral anticoagulants; RFCA, radiofrequency catheter ablation.
